# Role of Musclin in the Pathogenesis of Hypertension in Rat

**DOI:** 10.1371/journal.pone.0072004

**Published:** 2013-08-05

**Authors:** Ying-Xiao Li, Kai-Chun Cheng, Akihiro Asakawa, Ikuo Kato, Yuki Sato, Haruka Amitani, Namiko Kawamura, Juei-Tang Cheng, Akio Inui

**Affiliations:** 1 Department of Psychosomatic Internal Medicine, Kagoshima University Graduate School of Medical and Dental Sciences, Kagoshima, Japan; 2 Department of Bioorganic Chemistry, Faculty of Pharmaceutical Sciences, Hokuriku University, Kanazawa, Japan; 3 Department of Medical Research, Chi-Mei Medical Center, Tainan, Taiwan; The University of Manchester, United Kingdom

## Abstract

Musclin is a novel skeletal muscle-derived secretory factor found in the signal sequence trap of mouse skeletal muscle cDNAs. Musclin possesses a region homologous to the natriuretic peptide family. Thus, musclin is found to bind with the natriuretic peptide clearance receptors. However, the role of musclin in vascular regulation remains unclear. In this study, we aim to investigate the direct effect of musclin on vascular tone and to analyze its role in hypertension using the spontaneously hypertensive rats (SHR). In aortic strips isolated from SHR, musclin induced contractions in a dose-dependent manner. We found that the musclin-induced vasoconstriction was more marked in SHR than in normal rats (WKY). Moreover, this contraction was reduced by blockade of natriuretic peptide receptor C using the ab14355 antibody. Therefore, mediation of the natriuretic peptide receptor in musclin-induced vasoconstriction can be considered. In addition, similar to the natriuretic peptide receptor, expression of the musclin gene in blood vessels was higher in SHR than in WKY. Injection of musclin markedly increased the blood pressure in rats that can be inhibited by anti-musclin antibodies. Musclin-induced vasoconstriction was more pronounced in SHR than in WKY as in its expression. Taken together, these results suggest that musclin is involved in blood pressure regulation. The higher expression of musclin in hypertension indicates that musclin could be used as a new target for the treatment of hypertension in the future.

## Introduction

Musclin is a novel muscle-derived secretory peptide found in the signal sequence trap of mouse skeletal muscle cDNAs. Musclin mRNA was almost exclusively expressed in the skeletal muscle of rodents and obesity models [1]. The function of musclin has been described as responsive to insulin in vivo and inducing insulin resistance in vitro [2–4]. Furthermore, musclin is known to be a bone-active molecule that is highly expressed in cells of the osteoblast lineage of animals [5,6].

Musclin contains a region homologous to the members of the natriuretic peptide (NP) family, as well as a KKKR putative serine protease cleavage site, which is also characteristic of NP proteins [1]. The mammalian NP family comprises the atrial natriuretic peptide (ANP), B-type natriuretic peptide (BNP), and C-type natriuretic peptide (CNP). NP is a family of structurally related but genetically distinct hormones/paracrine factors that regulate blood volume, blood pressure, ventricular hypertrophy, pulmonary hypertension, fat metabolism, and long-bone growth [7]. Three single membrane-spanning NP receptors (NPRs) have been identified; NPR-A and NPR-B are linked to trans-membrane guanylyl cyclases, and NPR-C lacks intrinsic enzymatic activity and controls the local concentrations of NP through constitutive receptor-mediated internalization and degradation [5]. Previous studies reported that musclin binds with high affinity to NPR-C, but not to NPR-A or NPR-B, in a manner that is competitive with ANP [5,8].

The mechanistic involvement of NPR-C in the regulation of vascular functions has been documented. When physiologically activated, NPR-C seems to exert vascular protective effects, suggesting a direct antihypertensive role. Indeed, CNP/NPR-C signal has also been suggested as a fundamental pathway underlying the endothelium-derived hyperpolarizing factor-dependent regulation of vascular tone in rat mesenteric resistance arteries and in the coronary vasculature [9]. Therefore, musclin binding to NPR-C may regulate vascular tone locally and/or systemically.

However, little is known about the effect of musclin on vascular tone, especially in pathological conditions. In the present study, we investigated the reaction of musclin with aortic strips isolated from either the normal Wistar Kyoto rats (WKY) or the spontaneously hypertensive rats (SHR). In addition, a specific antibody was used to demonstrate the potential relationship between musclin and NPR-C. Further, we analyzed the differences in the gene expression of musclin and NPR-C between WKY and SHR. Finally, we injected musclin into normal rats to observe the increase in blood pressure and applied the musclin antibodies in SHR to find the recovery of blood pressure. Thus, in the present study, we identified the role of musclin in blood pressure regulation.

## Materials and Methods

### Musclin synthesis and preparation of drugs

Mouse musclin (SFSGFGSPLDRLSAGSVEHRGKQRKAVDHSKKRFGIPMDRIGRNRL-SSSRG) was synthesized through a solid-phase methodology by using 9-fluorenylmethyloxycarbonyl on an automated peptide synthesizer (Model Pioneer; Life Technologies, CA, USA). The crude peptide was purified by reverse-phase HPLC (Delta 600 HPLC system; Waters, MA, USA) on a Mightysil RP-18 GP column (2 × 25 cm; Kanto Chemical, Tokyo, Japan). The homogeneity of the purified peptide was confirmed by analytical HPLC, MALDI-TOF mass spectrometry (MS), and amino acid analysis.

### Experimental animals

Male SHR and age-matched WKY, weighing 320–340 g, were obtained from the National Animal Center (Taipei, Taiwan). They were maintained in a temperature-controlled room (25°C ± 1°C) under a 12:12 light-dark cycle (light on at 06:00 AM). Food and water were available ad libitum. All the animal procedures were performed according to the Guide for the Care and Use of Laboratory Animals of the National Institutes of Health. The animal experiments were approved by the Regional Ethics Committee for Animal Research in Chi-Mei Medical Center (Tainan, Taiwan) and Kagoshima University (Kagoshima, Japan).

### Preparation of isolated aorta strips

The rats were euthanized by decapitation under anesthesia with 3% isoflurane. We rapidly removed the aorta and placed the sample in oxygenated Krebs buffer (95% O_2_, 5% CO_2_). After dissection, the aorta was cut into strip segments approximately 5 mm long. The strips were then mounted on 37° C organ baths filled with 10 mL of oxygenated Krebs buffer (95% O_2_, 5% CO_2_) containing 135 mmol/L NaCl, 5 mmol/L KCl, 2.5 mmol/L CaCl_2_, 1.3 mmol/L MgSO_4_, 1.2 mmol/L KH_2_PO_4_, 20 mmol/L NaHCO_3_, and 10 mmol/L d-glucose (pH 7.4). Each preparation was connected to strain gauges (FT03; Grass Instrument Co, Quincy, MA, USA). Isometric tension was recorded by using a chart-software (MLS023, Powerlab; A D Instruments Pty Ltd., Bella Vista, New South Wales, Australia). The strips were mounted and allowed to stabilize for 1.5 h. The preparation was then gradually stretched to achieve an optimal resting tension of 0.5 g. The contractions were calculated as the percentage of the increase in vascular tone induced by treatment with potassium chloride (50 mmol/L).

### Aortic contraction caused by musclin

After the tension had stabilized, the aortic strips were exposed to musclin at various concentrations (0.01–10 nmol/L), with a wait time of 15–20 min between each musclin dosing. Then, the increase in tonic contraction (vasoconstriction) was evaluated. Once the sample stabilized, oxygenated Krebs buffer was replaced, and then potassium chloride (50 mmol/L) (Sigma-Aldrich, St Louis, MO, USA) was added as a positive control. Vasoconstriction was expressed as the percentage of potassium chloride (50 mmol/L)-induced contraction, while the concentration–response curve was generated in a cumulative fashion.

### Removal of endothelium

To exclude a possible role of the endothelium in musclin-induced vasoconstriction, the tests were conducted in endothelium-denuded preparations. The endothelium was removed by gently rubbing it against the teeth of a pair of forceps. Successful removal of the endothelium was confirmed by histological identification and failure of 1 µmol/L acetylcholine to relax the rings that had been pre-contracted with potassium chloride as described previously [10].

### Anti-NPR-C antibody on musclin-induced aorta contraction

Aortic strips were exposed to anti-NPR-C antibody (ab14355) at dilutions of 1:10000, 1:5000, and 1:2500 (ABCAM, Cambridge, MA, USA) for 15 min before addition of musclin (0.01 nmol/L). The changes in vasoconstriction of musclin-treated samples were compared with those in vehicle (distilled water)-treated controls. cANP or ANP_4-23_ (Phoenix Pharmaceuticals, Inc., Burlingame, CA, USA), a ring deleted version of ANP, was used as a specific NPR-C receptor agonist. The specificity of anti-NPR-C antibody antibody was characterized in A7r5 cells by using the calcium influx assay method in our preliminary experiments. Since cANP has the similar molecular structure with musclin, we also compared the effect of cANP on vasoconstriction in SHR and WKY. Aortic strips were exposed to cANP at concentration of 0.1, 1, 10, 100 nmol/L, and the changes in vasoconstriction of cANP-treated samples in SHR were compared with those in WKY.

### Cell line and culture conditions

Rat cell line for vascular smooth muscle cells (A7r5 cells) (BCRC, Hsinchu, Taiwan) were cultured in RPMI-1640 medium (Gibco BRL, Paisley, Scotland) supplemented with 10% fetal calf serum (FCS) (Biologic Industries, Kibbutz Beit Haemek, Israel), penicillin (100 IU/mL), streptomycin (100 mg/mL) (Sigma, St. Louis, MO, USA), and amphotericin B (2.5 mg/mL, Gibco). The cells were trypsinized (trypsin used was purchased from Gibco) and subcultured once a week, and the medium was changed every 3–4 days. For the experiments, the cells were seeded on round (25 mm diameter) sterile glass coverslips and cultured for 48–72 h in RPMI-1640 medium supplemented as mentioned above.

### Measurement of intracellular calcium concentrations

The changes in the intracellular calcium concentration were detected using the fluorescent probe fura-2 [11]. A7r5 cells were placed in buffered physiological saline solution (PSS) containing 140 mM NaCl, 5.9 mM KCl, 1.2 mM CaCl_2_, 1.4 mM MgCl_2_, 11.5 mM glucose, 1.8 mM Na_2_HPO_4_, and 10 mM Hepes-Tris; next, 5 µM fura-2 was added to this solution; and then, the cells were incubated for 1 h in humidified atmosphere containing 5% CO_2_ and 95% air at 37° C. The cells were washed and incubated for further 30 min in PSS. The A7r5 cells were then inserted into a thermostated (37° C) cuvette containing 2 mL of calcium-free PSS and various doses of musclin or inhibitor as indicated. The fluorescence was continuously recorded using a fluorescence spectrofluorimeter (Hitachi F-2000, Tokyo, Japan). The values of intracellular calcium ([Ca^2+^]_i_) were calculated from the ratio R = F340/F380 by the formula [Ca^2+^]_i_ = K_d_B (R −R_min_)/(R_max_ − R), where K_d_ is 225 nM, F is the fluorescence measured at 340 nm and 380 nm, and B is the ratio of fluorescence of the free dye to that of the Ca^2+^-bound dye measured at 380 nm. R_max_ and R_min_ were determined in separate experiments by using musclin to equilibrate [Ca^2+^]_i_ with ambient [Ca^2+^] (R_max_) and adding 0.1 mM MnCl_2_ and 1 mM EGTA (R_min_). Background auto-fluorescence was measured in unloaded cells and was subtracted from all measurements.

### Determination of musclin-induced blood pressure changes in WKY and SHR

WKY and SHR were anesthetized with phenobarbital (50 mg/kg i.p.). We separately administered musclin (0.05 mg/kg) and anti-musclin antibody FL-133 (Santa Cruz Biotechnology, Santa Cruz, CA, USA) at 1:5000, 1:1000, or 1:500 dilutions to the tails of WKY and SHR by intravenous injection. Systolic blood pressure of anesthetized WKY or SHR was measured using the tail cuff method by a sphygmomanometer without animal heating (Muromachi Kikai Co., Ltd., Tokyo, Japan). Blood pressure was measured at 5-min intervals after intravenous injection of musclin. Each value was presented as the average of 3 independent readings.

### Western blot analysis

The expression of musclin and NPR-C was examined in the membrane protein fraction obtained from aorta homogenates. Aorta homogenates were centrifuged at 1,000 × *g* for 10 min at 4° C, and the pellets were resuspended in ice-cold homogenized buffer. Then, the blots were separately developed using anti-NPR-C antibodies (ab14355) (ABCAM, Cambridge, MA, USA) and anti-musclin antibodies (FL-133). The intensity of the blots incubated with actin (Millipore) was used as internal control. Blots were incubated with the appropriate peroxidase-conjugated secondary antibodies. After removal of the secondary antibody, the blots were washed and developed by autoradiography using the ECL-Western Blotting System (Amersham Corp., Braunschweig, Germany). The densities of the obtained immunoblot bands at 48 KDa (outer membrane receptor [OMR]) and 43 KDa (actin) were quantified using the Gel-Pro Analyzer software 4.0 (Media Cybernetics, Silver Spring, MD, USA). In addition, the expression of NPR-C in the A7r5 cell line was detected by western blot analysis in the same manner.

### Statistical analysis

All the values are presented as mean ± standard error of mean (SEM) for a given number of animals or samples. Statistical analysis was performed in SPSS. The statistically significant difference was evaluated using one-way ANOVA with Dunnett’s test compared against no antibody for each strain in the musclin-induced vasoconstriction and the inhibitory effect of the anti-NPR-C antibody on the calcium influx in A7r5 cells. 2-way repeated measures ANOVA for each strain in others. Musclin and NPR-C expression in aortic tissue used Student t-test. The difference was compared at the significance level of 2α=0.05 in ANOVA. A *p*-value of 0.05 was considered statistically significant in Student t-test.

## Results

### Effect of musclin on aortic strips isolated from rats

**Figure 1:**
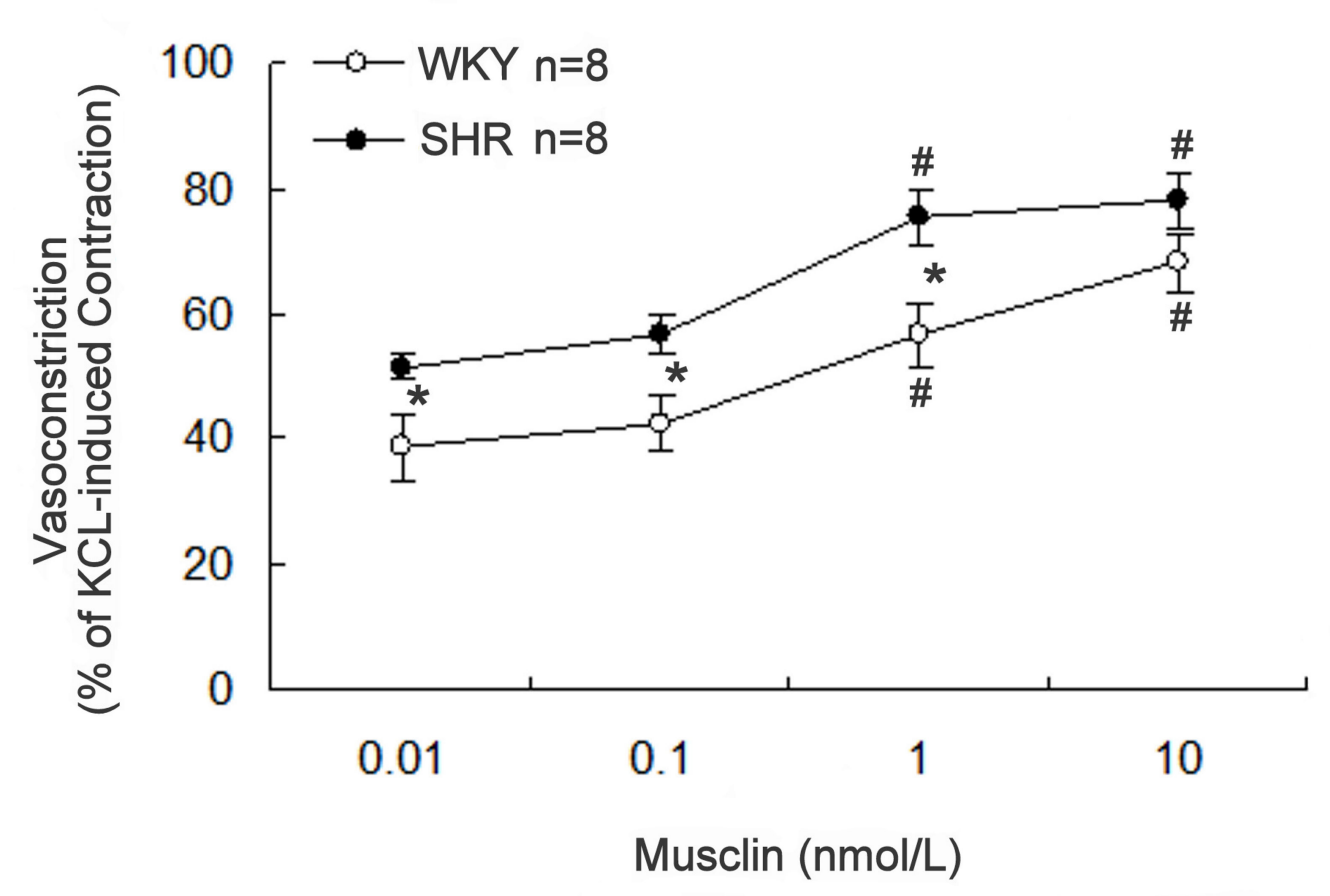
Effect of musclin on aortic strips isolated from rats. Concentration-dependent vasoconstriction induced by musclin in aorta strips isolated from SHR (black line) or WKY (broken line). Vasoconstriction is expressed as the percentage of potassium chloride (50 mmol/L)-induced contraction. Data represent the mean ± SEM of 8 animals. *Significance as compared with WKY and ^#^ significance as compared with the group treated with musclin at 0.01 nmol/L using 2-way repeated measures ANOVA for each strain.

Vasoconstriction was induced in a concentration-dependent manner by musclin (0.01–10 nmol/L) in the aortic strips isolated from WKY or SHR. The response to musclin in SHR aorta was stronger than that in WKY (Figure 1). Aortic strips treated with potassium chloride (50 mmol/L) were used as controls to simulate contraction under physiological conditions. The effects of musclin were deleted by washing the strips with no-musclin buffer, and the results could be reproduced by re-treatment with musclin.

### Effect of NPR-C blockade on musclin-induced vasoconstriction

**Figure 2:**
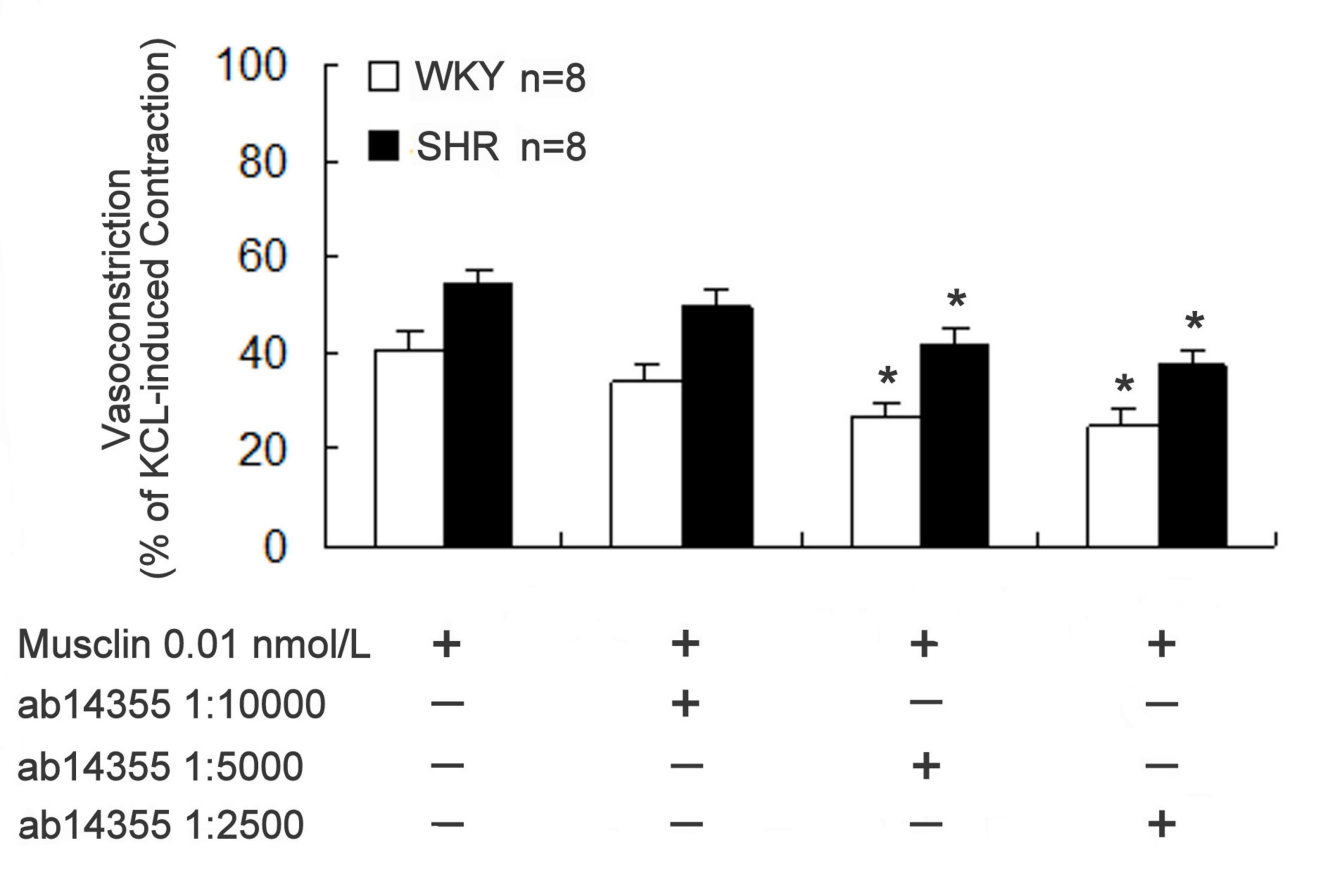
Effect of NPR-C blockade on musclin-induced vasoconstriction. Inhibitory effect of the antibody (ab14355) specific to NPR-C on vasoconstriction induced by musclin (0.01 nmol/L) in aorta strips isolated from spontaneously hypertensive rats (SHR). In the presence of the indicated concentration of ab14355, the responses to musclin were evaluated. Vasoconstriction induced by musclin was expressed as the percentage of contraction induced by potassium chloride (50 mmol/L). Data represent the mean ± SEM of 8 animals. *Significance as compared with the vehicle-treated control (without ab14355) using one-way ANOVA with Dunnett’s test compared against no antibody.

To elucidate the relationship between musclin and NPR-C, the aortic strips were pre-treated with ab14355 anti-NPR-C antibody at dilutions of 1:10000, 1:5000, and 1:2500 before treatment with buffer containing 0.01 nmol/L musclin. This pre-treatment led to a concentration-dependent attenuation of the musclin-induced vasoconstriction (Figure 2). The vasoconstriction induced by potassium chloride (50 mmol/L) was reduced to 98.6% ± 4.3% (n = 8) by the anti-NPR-C antibody at 1:2500 dilution without significant difference (P > 0.05) with the vehicle-treated control (97.6% ± 3.9%, n = 8). However, the treatment with control IgG (GE healthcare, Tokyo, Japan) at 1:2500 dilution did not significantly change the musclin-induced vasoconstriction (53.82 ± 3.27%, n=8) in aortic strips from SHR.

### Role of the endothelium in musclin-induced vasoconstriction

Successful removal of the endothelium was confirmed in each strip. Since the removal of the endothelium did not cause any effect, musclin-induced vasoconstriction was calculated as the percentage of potassium chloride-induced contraction (data not shown). In SHR aortic rings without endothelium, musclin-induced vasoconstriction was 82.16% ± 1.96%, which was not significantly different from that in aortic rings with endothelium (82.58% ± 2.62%, P > 0.05; n = 8). Similar results were observed in aortic rings isolated from WKY, for which vasoconstriction in the presence and absence of endothelium was 61.85% ± 0.83% and 63.96% ± 1.28% (n = 8, P > 0.05), respectively.

### Presence of NPR-C in A7r5 cell line

**Figure 3:**
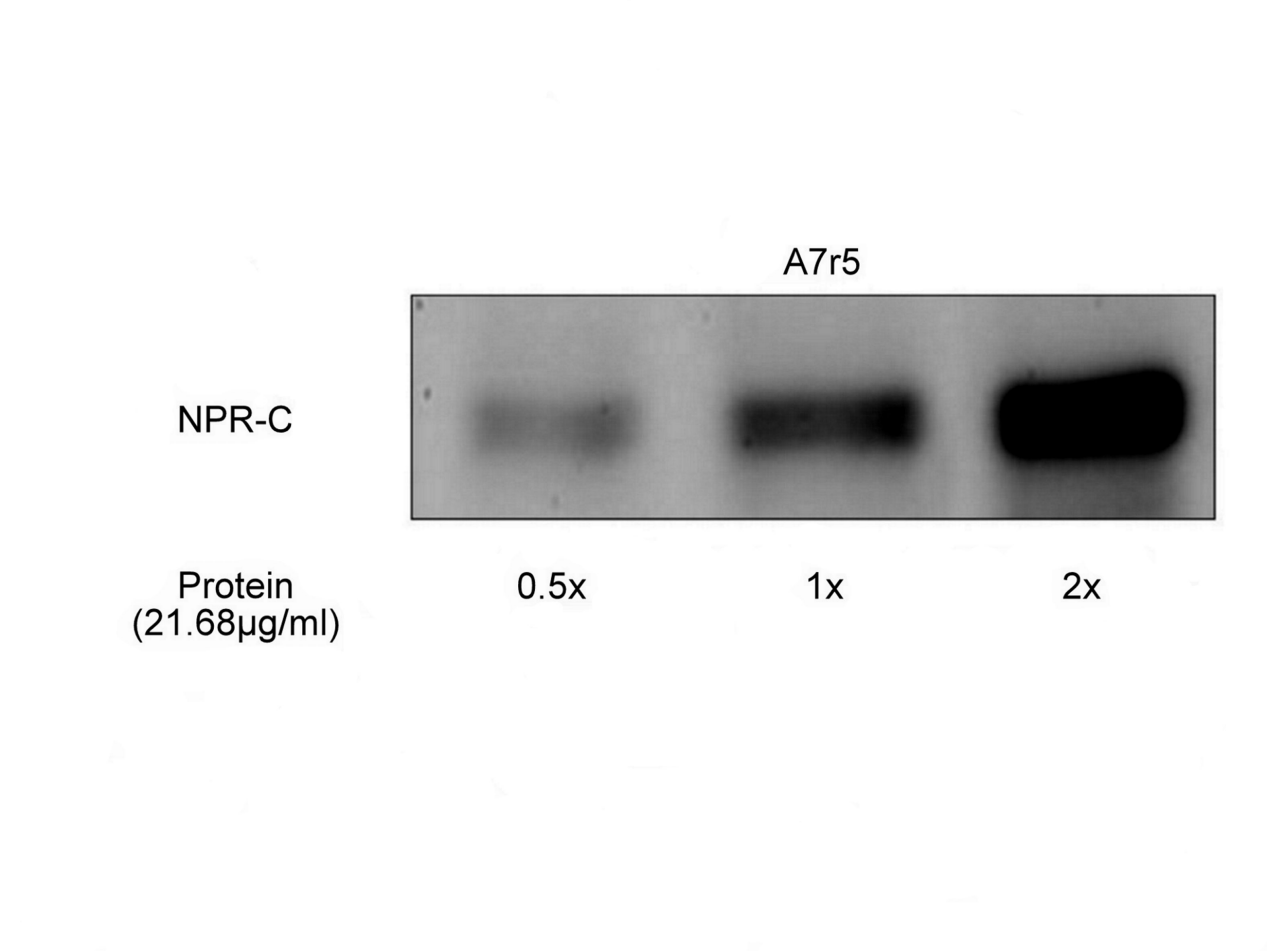
Western blot analysis of the membrane fraction from A7r5 cells showing the expression of natriuretic peptide receptor C (NPR-C). The membrane fraction of A7r5 cells was probed with anti-NPR-C antibodies. The expression of NPR-C increased with increase in the amount of the loaded membrane protein (21.68 µg/mL) from lane 1 to 3. Immunoblot analyses were repeated 3 times, and the results obtained were similar.

Western blot analysis demonstrated the expression of NPR-C in the A7r5 cell line. The expression of NPR-C increased with increasing membrane protein amounts, and this increase was in multiples of 21.68 µg/mL (Figure 3).

### Changes in [Ca^2+^]_i_ caused by musclin in A7r5 cells

**Figure 4:**
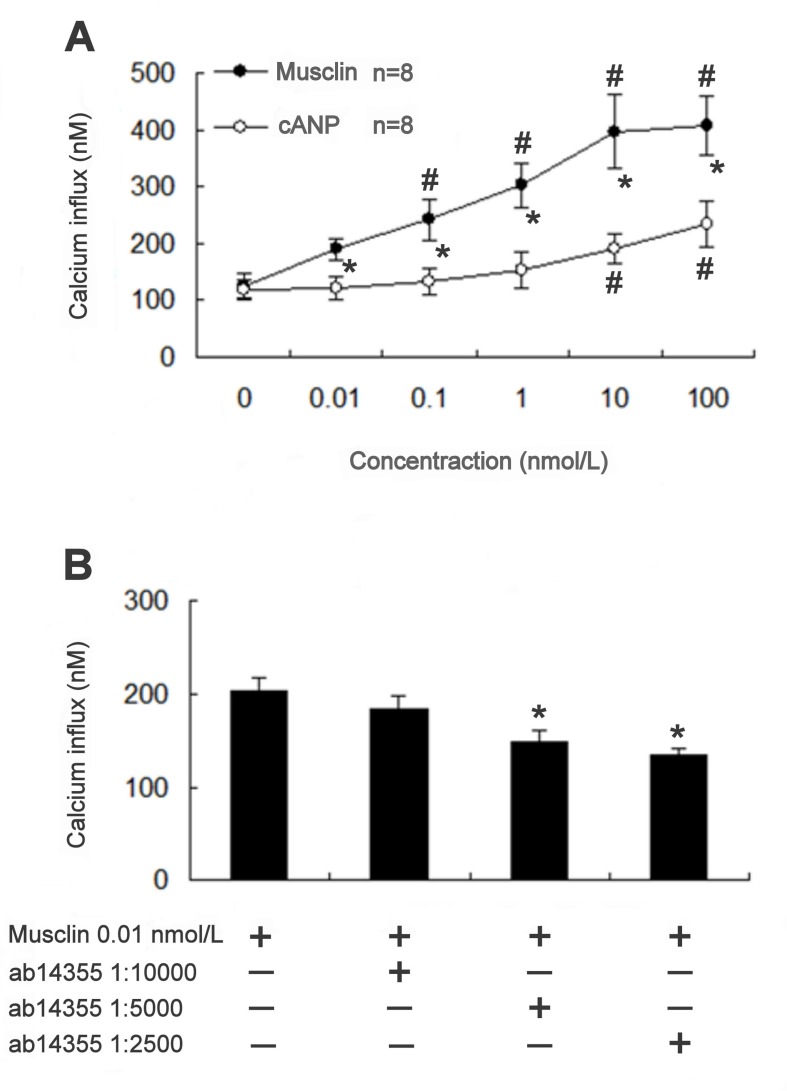
Changes in [Ca^2+^]_i_ caused by musclin in A7r5 cells. (A) Increase of calcium influx induced by musclin and cANP in A7r5 cell. The intracellular Ca^2+^ levels determined by FURA-2 showed concentration-dependent increase in response to musclin and cANP, and the vehicle-treated group was taken as control. *Significance as compared with cANP and ^#^ difference as compared with the control group using 2-way ANOVA. (B) Inhibitory effect of the anti-NPR-C antibody (ab14355) on the increase in calcium influx induced by musclin (0.01 nmol/L) in A7r5 cell. The changes in the calcium influx induced by musclin were evaluated in the presence of the indicated concentrations of ab14355. Results, expressed as mean ± SEM, were obtained from 8 independent experiments. * Significance as compared against no antibody group using one-way ANOVA with Dunnett’s test.

We tested the role of [Ca^2+^]_i_ in vasoconstriction caused by cANP and musclin. After incubation with musclin, the concentration of [Ca^2+^]_i_ in A7r5 cells significantly increased in a concentration-dependent manner. cANP also induced a similar response in A7r5 cells (Figure 4A). To determine the relationship between musclin and NPR-C, ab14355 anti-NPR-C antibody was pre-treated at 1:10000, 1:5000, 1:2500 dilutions in the musclin-contained (0.01 nmol/L) buffer. This analysis revealed that the pre-treatment induced a concentration-dependent attenuation of the calcium influx, which was increased by musclin in A7r5 cells (Figure 4B). cANP increased calcium influx into A7r5 cells in a concentration-dependent manner and the maximal action produced was half of that induced by musclin at 10 nmol/L. This action of 100 nmol/L c-ANP was completely blocked by the anti-NPR-C antibody at 1:2500 dilution (not shown). However, treatment with the same dilution of the anti-NPR-C antibody failed to interfere with the action of potassium chloride (30 mmol/L). Thus, the specificity of the ab14355 antibody toward NPR-C was verified.

### High expression of musclin in SHR

**Figure 5:**
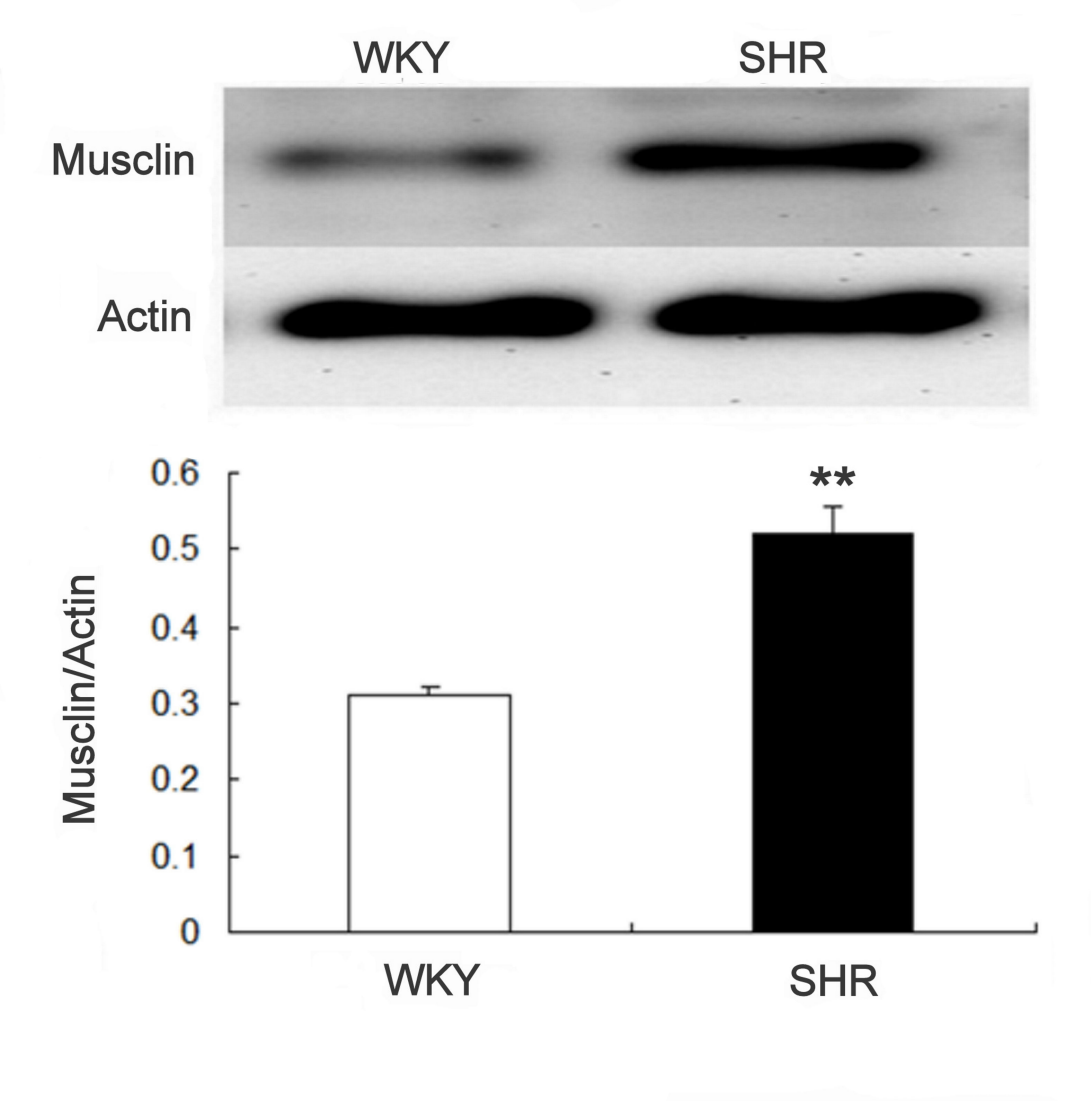
Difference in musclin expression in aortic tissues of WKY and SHR. The upper panel shows a representative picture of the expression in 2 experiments. Data in the lower panel represent the mean ± SEM of 8 animals. **P < 0.01 compared with WKY using Student t-test.

Musclin expression in aortic tissue was more marked in SHR than in WKY (Figure 5). The difference between the 2 groups after quantification of protein levels is shown in the lower panel.

### High expression of NPR-C in SHR

**Figure 6:**
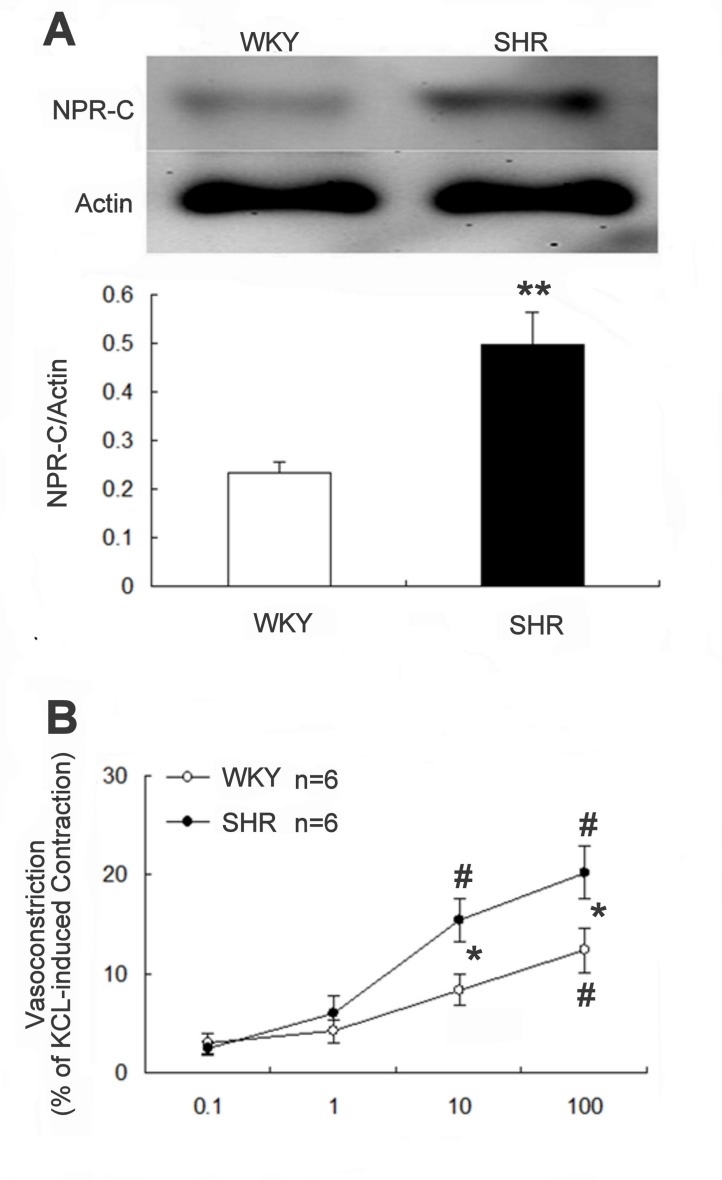
Difference in NPR-C expression and cANP-induced constriction in aortic tissues of WKY and SHR. (A) Difference in the NPR-C expression in aortic tissues of WKY and SHR. The upper panel shows a representative picture of the expression in 2 experiments. Data in the lower panel represent the mean ± SEM of 8 animals. **P < 0.01 compared with WKY using Student t-test. (B) Effect of cANP-induced constriction in aortic tissues of SHR and WKY (n = 6). *Significance as compared with WKY and ^#^ significance as compared with the group treated with cANP at 0.1 nmol/L using 2-way repeated measures ANOVA.

The gene expression of NPR-C in aortic tissue of SHR was higher than that in WKY. The difference between the 2 groups after quantification of protein levels is shown in the lower panel (Figure 6A). Also, the agonist of NPR-C receptor (cANP at 100 nmol/L) induced vasoconstriction in isolated aortic rings was more significant in SHR than in WKY (20.2 ± 2.6% vs. 12.3 ± 2.3% of potassium chloride-induced response, n = 6, P<0.05) (Figure 6B).

### Increase of blood pressure by musclin in normal *rats*

**Figure 7:**
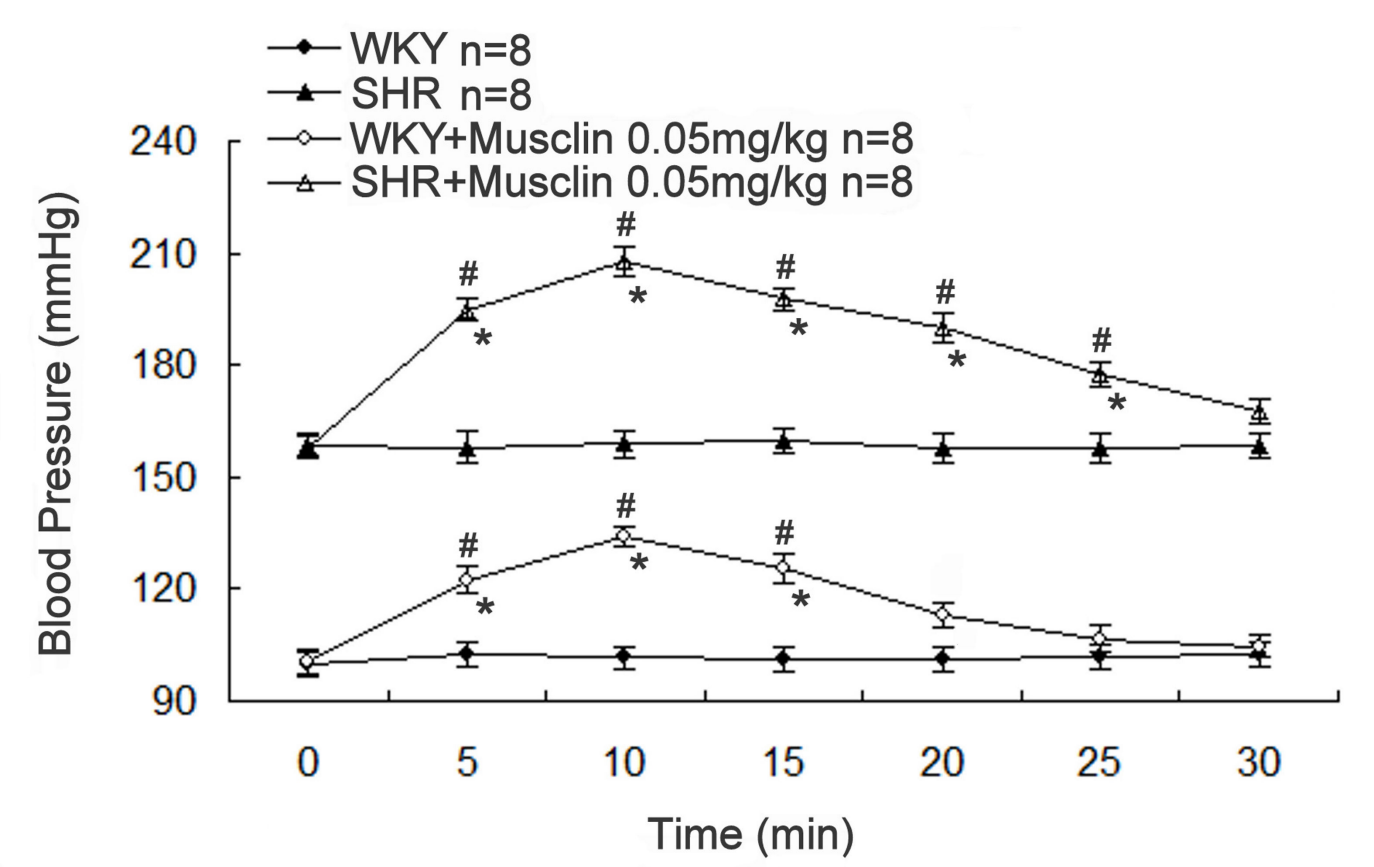
Changes of systolic blood pressures in WKY and SHR. The blood pressure in WKY and SHR were observed after intravenous injection of musclin (0.05 mg/kg). The systolic blood pressures were monitored using the tail-cuff method. Data are presented as mean ± SEM of 8 animals. *Significance compared with that without the treatment of musclin at the same point and ^#^ significance as compared with that at 0 minute using 2-way repeated measures ANOVA for each strain.

We further examined the effect of musclin on blood pressure in rats. Both WKY and SHR anesthetized rats showed a marked increase in the systolic blood pressure after musclin (0.05 mg/kg) treatment via injection into the tail vein (Figure 7). The increase in systolic blood pressure was significantly higher in SHR than in WKY (P < 0.05).

### Decrease of blood pressure by musclin antibodies in SHR

**Figure 8:**
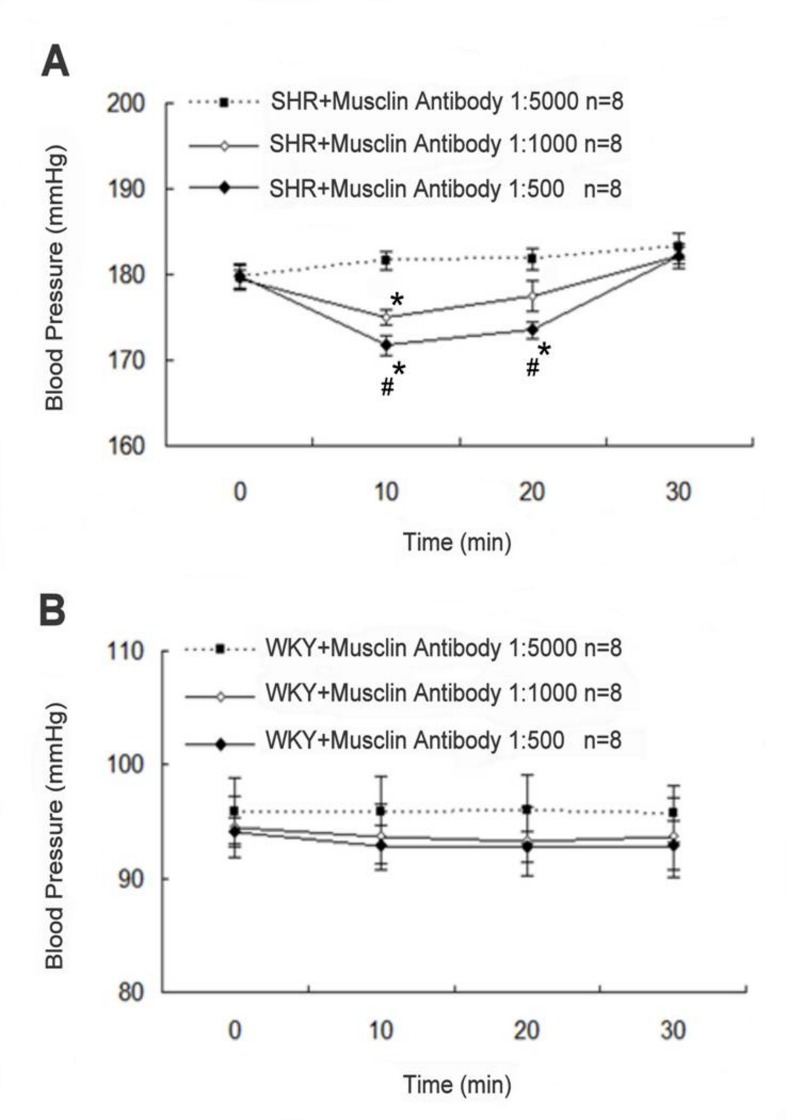
Changes of blood pressure by musclin antibodies in SHR. (A) Changes in the blood pressure of SHR or (B) WKY observed after intravenous injection of anti-musclin antibodies. Anti-musclin antibodies at the indicated dilutions were injected into rats. The blood pressure at 0 min was taken as basal control. Blood pressure values are expressed as mean ± SEM (n = 8). *Significance compared with that treated with antibody 1:5000 at the same time point and ^#^ significance compared with that at 0 minute using 2-way repeated measures ANOVA for each strain.

The anti-musclin antibody FL-133 was used to investigate the effect of musclin on blood pressure in SHR. The solutions of FL-133 at 1:5000, 1:1000, or 1:500 dilutions were administered to SHR via intravenous injection. FL-133 at 1:5000 dilution did not induce a change in the blood pressure, and it was therefore taken as the control. Differently from the control, FL-133 at 1:1000 dilution decreased the blood pressure in SHR at 10 min after the injection (P < 0.05, n = 8) (Figure 8A). FL-133 at 1:500 dilution lowered the blood pressure in SHR more markedly at 10 min and 20 min after the injection (P < 0.01, n = 8). However, the blood pressure recovered within 30 min after the injection of FL-133 in all concentrations. In contrast, similar treatment with FL-133 at the same dilutions failed to modify the blood pressure in WKY (Figure 8B). Otherwise, treatment with control IgG at 1:500 dilution made no change of blood pressure in SHR (201.6 ± 10.9 mmHg; P>0.05, n=8).

## Discussion

In the present study, we found that musclin induced vasoconstriction in aortic strips isolated from WKY or SHR in a concentration-dependent manner. The response in SHR was more marked than in WKY. In addition, we used the ab14355 anti-NPR-C antibody to abolish the vasoconstriction caused by musclin in order to highlight the mediation of NPR-C. The first (88LDRL91) and second (117MDRI120) NP-homologous regions of musclin are responsible for the cooperative high-affinity binding to NPR-C [3]. The binding of musclin with NPR-C has been demonstrated by assessing the competition with ANP [3]. Furthermore, the binding of NPR-C with musclin seems to be a regulatory pathway governing vascular tone. However, as shown in Figure 2, the highest concentration of the anti-NPR-C antibody used in this study decreased the vasoconstriction induced by musclin only by 20% and could not completely reverse musclin effect. Therefore, other mechanisms for musclin could be involved. The endothelium is generally recognized to play a key role in the regulation of vascular tone. However, in the present study, vasoconstriction induced by musclin was not influenced by removal of the endothelium. Hence, the role of the endothelium in the musclin-induced contraction of the aorta seems negligible.

The vasoconstriction induced by musclin appeared to be calcium-dependent. The intracellular signals linked with the NPR-C receptor have been demonstrated in cardiovascular tissues and cells [11]. Recently, it has been reported that ANP interacts with NPR-C in atria, ventricles, and the aorta to activate Ca^2+^-loaded calmodulin and endothelial nitric oxide synthase (eNOS) through the Gi protein [12]. In particular, impairment of the eNOS response to ANP has been observed in SHR as compared to normotensive WKY, thus suggesting that dysfunctions in the NPR-C signaling pathway, which leads to the reduced NO availability, may contribute to the maintenance of hypertension in SHR [13]. Musclin-induced increase in the concentration of intracellular calcium was observed in the widely used cultured vascular cells A7r5 [14]. A7r5 cells have been identified to contain NPR-C by radioligand binding analysis [15]. In the present study, we used Western blot analysis to confirm this view. Also, we used cANP to look at the increase of intracellular calcium in a way as that induced by musclin in A7r5 cells.

In SHR, both musclin and NPR-C expressions were higher than in WKY. The precise role of NPR-C in the development of hypertension is still unclear. NPR-C mRNA is found in atrial tissues, mesentery, placenta, lungs, kidneys, venous tissue [16,17], and in aortic smooth muscle and aortic endothelial cells [18]. Studies on mice with NPR-C loss-of-function mutations indicate that the main role of NPR-C is to clear natriuretic peptides from the circulation or extracellular milieu through receptor-mediated internalization and degradation [19]. Additionally, a whole-body NPR-C knockout mouse [20] shows profound skeletal abnormalities—consistent with the role of NPs in bone formation—and approximately 50% of the homozygous knockouts died before weaning. Interestingly, none of the homozygous animals with NPR-C mutations, which negatively affected any known natriuretic peptide response, displayed NPR-C loss-of-function. On the contrary, these animals displayed phenotypes associated with exaggerated NPR-A and NPR-B actions, such as hypotension and gigantism, respectively. These data suggest that in mice, NPR-A or NPR-B mediate the known effects of natriuretic peptides [7]. However, it is also possible that NPR-C mediates some unknown natriuretic peptide functions.

The main role of NPR-C is known to clear natriuretic peptides which would decrease blood pressure [19]. The possible role of NPR-C in the regulation of blood pressure relating to the development of hypertension has been mentioned in previous report [21]. The concentration of NPR-C is high in the vasculature of several animal models of hypertension and the observed response to pressure overload is likely a physiological response to increased vascular stress [22–24]. A study of tissue-specific distribution of NPR-C expression revealed that the concentration of NPR-C in the kidney of SHR is higher than that in the kidney of WKY [25]. Our findings in aorta are consistent with it.

The maximal vasoconstriction induced by the highest concentration of musclin (10 nmol/L) did not differ between SHR and WKY. In addition, the anti-NPR-C antibody could only induce a partial inhibition of vasoconstriction induced by musclin. It seems that only a part of musclin could bind with NPR-C to induce partial activation of NPR-C. Since musclin is not a specific ligand of NPR-C, it is hard to follow the traditional ligand-receptor relationship. Furthermore, the vascular-wall abnormalities in SHR arteries may lead to less efficient vessel contraction than that in the WKY samples [26]. Taken together, these data suggest that an incomplete matching of the maximal vascular contraction with increased NPR-C concentration in SHR and WKY is thus possible.

In the present study, we found a higher expression of musclin in SHR that has not been mentioned before. Moreover, the blood pressure was raised by injection of musclin into the rats. The decrease of blood pressure in SHR following anti-musclin antibody treatment showed the role of endogenous musclin in mediating the blood pressure regulation in SHR. However, the highest dose of the anti-musclin antibody showed a modest inhibition but not a complete blockade in the blood pressure of SHR. Therefore, other factors that might influence blood pressure regulation in SHR should be concerned and it needs to be investigated in the future. It seems necessary to generate tissue-specific musclin knockouts for the proper assessment of the functional role of musclin. Nevertheless, musclin really mediates blood pressure regulation, and the role of musclin in the pathogenesis of hypertension, at least in part, is identified in the present study using SHR.

In the in vitro studies, we observed musclin could induce vasoconstriction in aortic strips and increase the calcium concentrations in A7r5 cells. Because both were obtained without natriuretic peptides involved, it is suggested that musclin could trigger blood vessel contraction. In in vivo, blood pressure was raised by the injection of musclin into rats, but this action of musclin was only partially reduced by NPR-C antibody ab14335. Thus, musclin may have its own specific receptor and also could partially activate NPR-C. However, musclin competes effectively with NPs for binding to NPR-C sites and lead to increase in local concentrations of natriuretic peptides, which would be supposed to decrease blood pressure [20]. The possible explanation is that blood pressure elevation by musclin such as that in SHRs is strong enough to compensate the decreased blood pressure induced through natriuretic peptides. This is consistent with previous reports that plasma ANP was markedly raised in SHR without decrease in blood pressure [27,28]. The different vasoconstrictive potency of cANP in aortic strips of SHR and WKY was observed in the absence of endogenous natriuretic peptides and indicates NPR-C signaling is pathological in SHR. Taken together, our results demonstrated that musclin has the ability to induce vasoconstriction after binding with NPR-C and others, thereby increasing the blood pressure in rats. To the best of our knowledge, this is the first report on the role of musclin in blood pressure regulation. Musclin may be a new pharmacological target in the treatment of hypertension.
